# Metformin Attenuates Inflammatory Responses and Enhances Antibody Production in an Acute Pneumonia Model of *Streptococcus pneumoniae*

**DOI:** 10.3389/fragi.2022.736835

**Published:** 2022-04-29

**Authors:** Grace C. Lee, Alvaro G. Moreira, Cecilia Hinojosa, Raymond Benavides, Caitlyn Winter, Audrey C. Anderson, Chang-Jui Chen, Noemi Borsa, Gabrielyd Hastings, Cody A. Black, Sarah M. Bandy, Alexander Shaffer, Marcos I. Restrepo, Sunil K. Ahuja

**Affiliations:** ^1^ College of Pharmacy, The University of Texas at Austin, Austin, TX, United States; ^2^ Pharmacotherapy Education and Research Center, School of Medicine, The University of Texas Health Science Center at San Antonio, San Antonio, TX, United States; ^3^ The Foundation for Advancing Veterans’ Health Research, San Antonio, TX, United States; ^4^ Veterans Administration Research Center for AIDS and HIV-1 Infection and Center for Personalized Medicine, South Texas Veterans Health Care System, San Antonio, TX, United States; ^5^ Department of Pediatrics, The University of Texas Health Science Center at San Antonio, San Antonio, TX, United States; ^6^ Department Pulmonary Diseases and Critical Care Medicine, The University of Texas Health Science Center at San Antonio, San Antonio, TX, United States; ^7^ South Texas Veterans Health Care System, San Antonio, TX, United States; ^8^ Department of Pathophysiology and Transplantation, University of Milan, Milan, Italy; ^9^ Internal Medicine Department, Respiratory Unit and Adult Cystic Fibrosis Center, Fondazione IRCCS Ca’ Granda Ospedale Maggiore Policlinico, Milan, Italy; ^10^ Department of Medicine, The University of Texas Health Science Center at San Antonio, San Antonio, TX, United States; ^11^ Department of Microbiology, Immunology and Molecular Genetics, The University of Texas Health Science Center at San Antonio, San Antonio, TX, United States

**Keywords:** *Streptococcus pneumoniae*, vaccine response, metformin, aging, pneumonia

## Abstract

Metformin may potentially reverse various age-related conditions; however, it is unclear whether metformin can also mitigate or delay the deterioration of immunological resilience that occurs in the context of infections that are commonly observed in older persons. We examined whether metformin promotes the preservation of immunological resilience in an acute *S. pneumoniae* (SPN) infection challenge in young adult mice. Mice were fed metformin (MET-alone) or standard chow (controls-alone) for 10 weeks prior to receiving intratracheal inoculation of SPN. A subset of each diet group received pneumococcal conjugate vaccine at week 6 (MET + PCV and control + PCV). Compared to controls-alone, MET-alone had significantly less infection-associated morbidity and attenuated inflammatory responses during acute SPN infection. Metformin lowered the expression of genes in the lungs related to inflammation as well as shorter lifespan in humans. This was accompanied by significantly lower levels of pro-inflammatory cytokines (e.g., IL6). MET + PCV vs. control + PCV manifested enhanced SPN anticapsular IgM and IgG levels. The levels of SPN IgM production negatively correlated with expression levels of genes linked to intestinal epithelial structure among MET + PCV vs. control + PCV groups. Correspondingly, the gut microbial composition of metformin-fed mice had a significantly higher abundance in the Verrucomicrobia, *Akkermansia muciniphila,* a species previously associated with beneficial effects on intestinal integrity and longevity. Together, these findings indicate metformin’s immunoprotective potential to protect against infection-associated declines in immunologic resilience.

## Introduction

**FIGURE 1:**
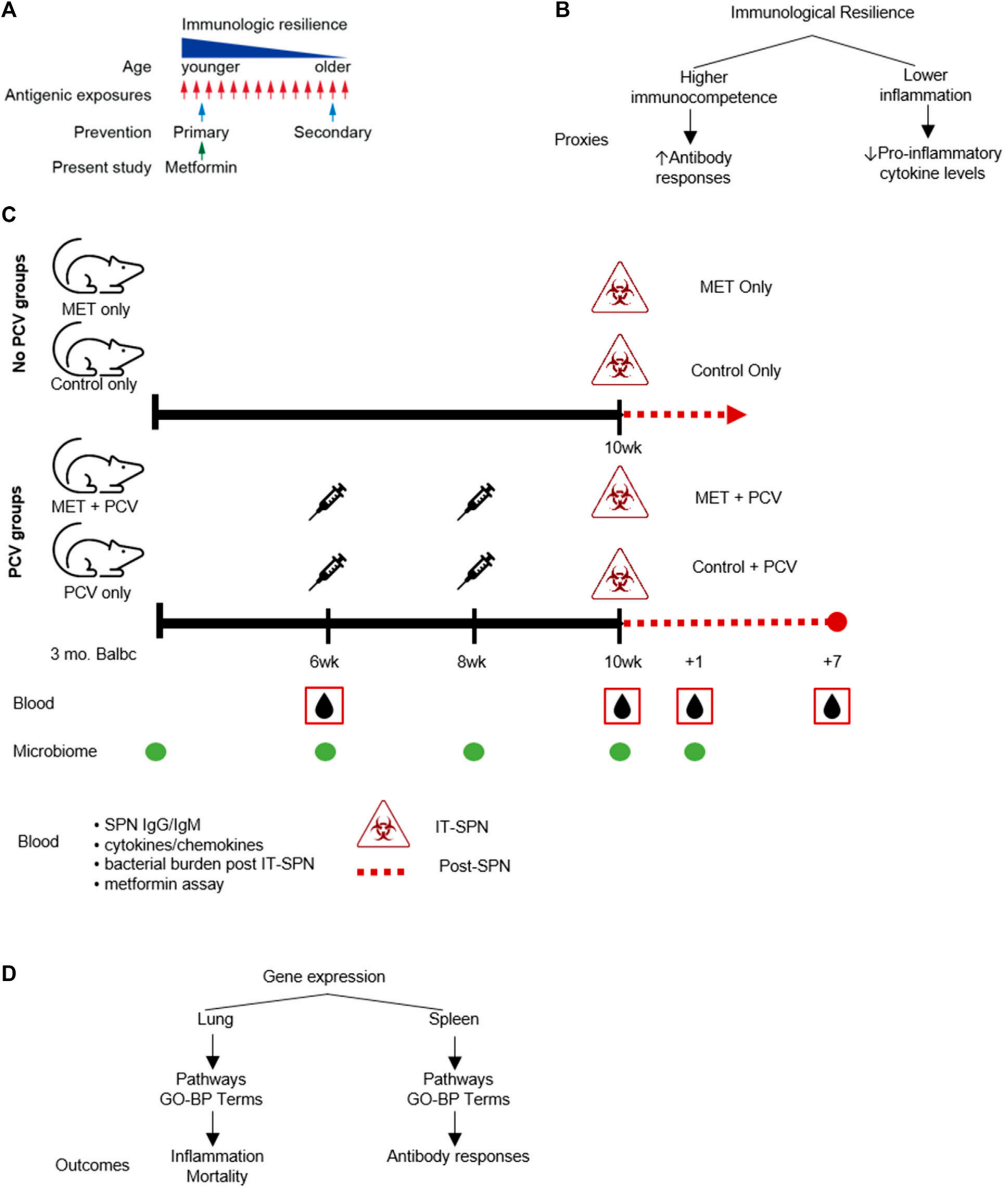
Study Schema. **(A)** Conceptual schema: prevention of immunologic resilience. **(B)** Conceptual schema of immunologic resilience and example proxies evaluated. **(C)** Study design. Four groups (*n* = 6 in each group) were designated as: 1) MET-alone, receiving metformin only; 2) control-alone, receiving only control diet; 3) MET + PCV, receiving both metformin and PCV13; and 4) control + PCV, receiving only PCV13 on a control diet. Top. No-pneumococcal conjugate vaccine (no-PCV) groups. Three-month old Balb/c female mice were fed AIN-93M rodent chow supplemented with either 0.1% w/w metformin (MET diet) or no MET (control diet). After 10 weeks on metformin or control diets, mice were challenged intratracheally (IT) with SPN and assessed for survival. All animals in no-PCV groups died on day 2 post SPN infection. Bottom. PCV groups. After 6 weeks on metformin or control diets, mice were administered PCV (two doses 2 weeks apart). At week 10, mice were inoculated IT with SPN. Mice were continued on MET or control diet until death or 7 days post infection. Blood samples were collected at week 6 prior to PCV, week 10 prior to SPN infection, and at +1 and +7 days post-SPN infection; SPN antibodies were measured at week 6 and 10 prior to infection. Fecal samples for microbiome analyses were collected at baseline, week 6, 8, 10, and post infection. **(D)** Comparative gene expression analysis was conducted in lung tissue and splenic cells. Differentially expressed genes between metformin and control groups were used to identify gene-ontologic biological processes (GO-BP terms) to evaluate for associations with inflammation and mortality (lung tissue) and antibody responses (splenic cells). PCV, pneumococcal conjugate vaccine; MET, metformin; SPN, *Streptococcus pneumoniae*; IT, intratracheal; GO-BP term, gene-ontologic biological processes.

The arc of aging-associated changes that influence immunologic health begins in early life (Lee et al., 2021). These changes can be linked to chronological aging, indexed to the passage of time, leading to time-based erosion of immunological health. Immunological health can also be shaped by factors that are not dependent on the passage of time (age) including the interaction between host genetic/epigenetic factors × environmental factors (e.g., infection). For example, infectious or inflammatory triggers may, at any age, reduce immunologic health, and the accumulation of these individual responses (starting from fecundation) can shape the extent of erosion of immunological health that persists throughout adulthood and older age (Figure 1A). For example, heightened inflammation due to environmental conditions at a younger age have associated with reduced vaccine responses, an indicator of impaired immunologic health (Kityo et al., 2018).

To begin to understand the time or age-independent factors that may negatively influence immunologic health, we recently conceptualized a process that we termed as *immunologic resilience* (Lee et al., 2021). We considered immunologic resilience to signify the capacity to preserve and/or restore higher levels of immunocompetence and concurrently suppress inflammation during antigenic exposure (e.g., infections) (Figure 1B) (Lee et al., 2021). A notable distinction from common definitions of “inflammaging”, is that the quotient of immunologic resilience reflects the conjoined effects of levels of immunocompetence plus inflammation (Figure 1B). We envisaged that repetitive antigenic exposures across the lifespan may erode immunological resilience, contributing to morbidity and mortality, as well as reduced vaccine responsiveness and recovery from stressors (e.g., infection, surgery, trauma) (Hadley et al., 2017; Kityo et al., 2018; Lee et al., 2021; Marconi et al., 2022). Conversely, persons who resist erosion of immunologic resilience during antigenic exposures manifest superior immunity-dependent outcomes, including longer lifespans. In accord with this thesis, we found that independent of age, persons preserving immunologic resilience resisted development of AIDS or severe COVID-19 (Lee et al., 2021). Moreover, we found that a subset of the gene expression signatures that associated with a survival advantage during COVID-19 also predicted a longevity advantage in persons from the Framingham Heart Study (i.e., without COVID-19) (Lee et al., 2021). Currently, anti-aging strategies are generally focused on reversal (i.e., secondary prevention) of traits observed among older persons and/or lifespan (secondary prevention; Figure 1A). However, it is conceivable that interventions that improve immunologic health from early adulthood may also have salutary effects in promotion of healthspan and lifespan.

Metformin is a first-line medication for individuals with or at risk for type-2 diabetes mellitus (T2DM) (Centers for Disease Control and Prevention, 2020). The immunomodulatory effects of metformin have prompted the consideration of repurposing metformin for immunoprotection against aging-related conditions (Le and Lee, 2019; Kulkarni et al., 2020). For example, metformin is associated with lower levels of markers of inflammation in persons with advanced forms of chronic inflammatory conditions (T2DM, autoimmune, cancer) (Pawelec, 2003; Berstein, 2012; Tomczynska et al., 2016; Christodoulou and Scorilas, 2017; Ursini et al., 2018). However, it is unclear whether metformin has utility for primary prevention (Figure 1A), i.e., mitigation of the decline in immunological resilience before there is a significant increase in the levels of inflammation or oxidative stress attributable to a high-grade antigenic challenge and/or the accumulative inflammatory burden of repetitive infections or immunological challenges experienced from early life.

Based on the guiding principles of primary prevention, herein, we examined whether metformin promotes immunological resilience during acute pneumococcal pneumonia in younger adult mice. We focused on this experimental model, as *Streptococcus pneumoniae* (SPN) is the most common bacterial cause of community-acquired pneumonia. Globally, SPN infections are a major cause of morbidity/mortality that disproportionally impacts immunosuppressed hosts (Feikin et al., 2000; Huss et al., 2009; Liu and Lee, 2019). To inquire into possible mechanisms by which metformin modulates immunologic resilience, we addressed these questions: does treatment of mice with metformin 1) before SPN infection dampen the inflammatory response after acute SPN infection? 2) associate with expression levels of genes signatures that we previously found to associate with survival in persons with COVID-19 and longevity in persons without COVID-19 (Lee et al., 2021)?, 3) enhance pneumococcal vaccine responsiveness (increase SPN antibody production) as a proxy of immunocompetence?, and 4) modulate the gut microbiota correlates of immunity?.

## Materials and Methods

### Animals, Diets and Cohorts

Three-month old Balb/c female mice were obtained from The Jackson Laboratories and housed in a temperature-controlled environment under pathogen free conditions. Mice were fed AIN-93M rodent chow supplemented with either 0.1% w/w metformin (MET diet) or no MET (Control diet) (Figure 1C). This dose was chosen based on the study by Martin-Montalvo et al. showing that MET (0.1% w/w in diet) was associated with extended lifespan and healthspan and that higher doses (1% w/w) were toxic (Martin-Montalvo et al., 2013). After 10 weeks on metformin or control diets, mice were challenged intratracheally with SPN as described previously and assessed for survival for 7 days post-infection (Figure 1C, top). To evaluate for SPN antibody responses (proxy for immunocompetence), mice in each diet group received pneumococcal vaccine after 6 weeks (PCV13; two doses 2 weeks apart; Figure 1C, bottom). MET diet was continued through end of experiment. Thus, four experimental groups were evaluated (Figure 1C); they were designated as: 1) MET-alone, receiving metformin only; 2) control-alone, receiving only control diet; 3) MET + PCV, receiving both metformin and PCV13; and 4) control + PCV, receiving only PCV13 on a control diet.

### Vaccine and Serological Immunogenicity

PCV13 (Prevnar 13 ®, Wyeth Pharmaceuticals) was from Pfizer Inc. and contains polysaccharides from pneumococcal 13 serotypes 1, 3, 4, 5, 6A, 6B, 7F, 9V, 14, 18C, 19A, 19F, and 23F, which are individually conjugated to a nontoxic diphtheria toxin cross-reactive material 197 protein. Vaccine formulation contains approximately 2.2 µg of each SPN serotypes 1, 3, 4, 5, 6A, 7F, 9V, 14, 18C, 19A, 19F, 23F saccharides, 4.4 μg of 6B saccharides, 34 µg CRM197 carrier protein, 100 µg polysorbate 80, 295 µg succinate buffer and 125 µg aluminum as aluminum phosphate adjuvant per 0.5 ml. Mice were immunized intramuscularly twice at 14 days intervals.

The efficacy of the vaccine was analyzed by quantification of total pneumococcal anti-capsular IgG and IgM antibodies via enzyme-linked immunosorbent assay (ELISA) before receipt of PCV and 2 weeks after the last PCV dose (Figure 1C).

### SPN Infection

All animal groups were infected with SPN 10 weeks after starting on metformin or control diet. *S. pneumoniae*, serotype 4, strain TGR4 was cultured in Todd-Hewitt broth or blood agar plates (Remel) at 37 C in 5% CO_2_. Cultures of TIGR4 were centrifuged and resuspended in sterile phosphate buffered saline (PBS). Mice were anesthetized with 2.5% vaporized isoflurane and then challenged intratracheally with approximately 10^5^ colony-forming units (CFU) in 100 µL of PBS. The infectious dose was verified by serially diluting the inoculum, plating, and extrapolating from colony counts following overnight incubation. Following infection, mice were housed in cages with additional bedding to help maintain warmth and food placed on the cage bottom. All non-vaccinated animals (Figure 1, top) died within 48 h of acute SPN pneumonia and sepsis at which time lung samples were collected and assessed. All vaccinated animals (Figure 1, bottom) survived the acute SPN pneumonia through 7 days. All animal experiments were according to approved Institutional Animal Care and Use Committee protocols at University of Texas Health San Antonio.

### Survival and Bacterial Burden

Pneumococcal disease severity was assessed by determining mortality rates after infection over a 7-day period. Survival was assessed by monitoring mice for the development of a moribund state, at which point mice were euthanized. Bacteremia was determined by aseptic collection of tail blood and measured every 24 h post-infection. Bacterial burden in the lungs was determined by aseptic removal of the lungs from euthanized mice and homogenizing the weighed lungs in PBS at time of death. Serial dilutions of both blood and lung homogenates were plated, incubated at 37°C in 5% CO_2_ for 24 h. The number of colonies were counted and expressed as log_10_ CFU per ml (blood) or per mg (lung tissue).

### Cytokines

Inflammatory cytokine and chemokine concentrations were measured in lung homogenates using the MILLIPLEX® MAP Mouse Cytokine/Chemokine Panel (CYTMAG-70K-PX32; Millipore Corp, Billerica, MA, United States) using the Luminex MAGPIX system. Freshly harvested mouse whole lung and liver tissue was flash frozen with liquid nitrogen and biobanked in liquid nitrogen. Lung tissue was kept on dry ice and homogenized on a Fast-Prep shaker for 2 min at room temperature. The tubes were centrifuged for 10 min at 10,000 rpm at 40°C. Three 200 µL supernatant volumes were collected and placed into micro tubes for biobanking at −80°C.

### Physiologic Evaluation

Animals were weighed daily with measurements of intake and output. After SPN infection, animals were monitored for one 7 days after SPN infection (Figure 1C). Oxygen saturation, body temperature, respiratory weight, and heart rates were assessed every 2 h after intratracheal administration of SPN for the first 48 h, then every 12 h.

### Histological Evaluation

Lung tissues were prepared for immunohistochemistry. Briefly, lung tissues were collected and inflated with buffered formalin. Two non-adjacent, 5 µm, lung sections from each mouse were stained with Hematoxylin and Eosin (H&E) at the UTHSA Pathology Core and examined with a digital pathology slide scanner. Lung sections were scored blindly on a scale of 0–5 according to the level of peribronchial and perivascular inflammation, neutrophil infiltration, and alveolar thickening and consolidation (Hinojosa et al., 2012; Hinojosa et al., 2020).

### Metformin LC/MSMS Assay

Metformin levels were evaluated in serum, liver, and lung tissues. After acetonitrile induced protein precipitation of the serum samples, metformin and the internal standard was chromatographed on a reverse phase XBridge Waters C18 (100 × 2.1 mm) analytical column. Quantitation was performed with a Waters Xevo TQD, a triple quadrupole mass spectrometer employing electrospray ionization technique and operating in multiple reactions monitoring (MRM) with positive ion mode. The total chromatograph run time was 5 min. The standard curves were linear (r^2^ > 0.99) over the concentration range of (5–3,000 ng/ml) for metformin.

### p-AMPK

The mouse AMPK ELISA kit (Biomatik, Fluidigm) was used to quantify p-AMPK activity on spleen cells according to manufacturer protocol. Biotinylated detection antibody specific for mouse AMPK and Avidin-Horseradish Peroxidase conjugate are added to each well and incubated. The optical density (OD) is measured spectrophotometrically at a wavelength of 450 nm.

### Gene Array

Gene expression analysis was performed in the lung tissue to identify gene pathways that associate with inflammation and mortality, and in splenic cells to identify pathways that correlate with SPN antibody levels (Figure 1D). Total RNA from lung tissue was extracted from flash frozen tissue using Total RNA Fatty and Fibrous Tissue kit (Bio-Rad). Freshly harvested mouse whole spleen tissue was homogenized and filtered with a 70 μm cell strainer, followed by a cell separation step with the use of Ficol-Paque Premium density gradient centrifugation. All density gradient isolated bands of cells were collected and stored in cryoprotective media. All cells were stored in liquid nitrogen until the time of assay analysis. RNA was extracted from 200 µL of each splenic sample using the Qiagen RNeasy Mini Kit according manufacturer’s instructions.

The synthesis of cDNA was isolated using the RT2 First Strand kit. Pathway specific gene expression was analyzed using the 1) RT2 Profiler PCR Array for Mouse Antibacterial Immune Response (Qiagen) for lung homogenates, and 2) Mouse Innate and Adaptive Immune Response (Qiagen) for splenic cells. Samples were run on the BioRad CFX-Touch Thermocycler according to manufacturer’s instructions. Data are reported as fold changes using the double‐delta CT formula.

### Gene Ontology Analysis

DAVID (v6.9) Huang et al., 2009 was used to perform GO and pathway analysis with genes whose expression levels were up/down regulated between study groups, or genes whose levels correlated with SPN antibody levels. The aggregated expression levels of genes comprising the GO-BP terms are reported as z-scores. To derive z-scores, the normalized expression of each gene is z transformed across all samples and then averaged (Lee et al., 2021).

### 16s rRNA Sequencing

Microbial DNA was extracted from fecal samples using the Qiagen DNeasy PowerSoil kit. Sequencing was conducted on the Illumina MiSeq instrument (Illumina, San Diego, CA) and analyzed using CLC Genomics Workbench and the CLC Microbial Genomics Module (Qiagen, Redwood City, CA). Sequences were clustered into operational taxonomic units (OTUs) and classified *via* Ribosomal Database Project’s Naïve Bayesian Classifier tool and referenced against the Greengenes database. A comparative analysis of abundance was conducted using UniFrac distances and visualized using principal coordinates analysis. To reduce noise, taxa with at least 0.1% combined abundance were included in the analyses.

### Statistical Analysis

Continuous data were expressed as mean with standard error of the mean and analyzed with Student’s t-test or Wilcoxon rank-sum test. Comparisons of more than two groups were with ANOVA or Kruskal–Wallis (followed by Dunn’s multiple comparisons test). Correlation analysis was with Spearman’s coefficient. A two-sided *p* < 0.05 was considered statistically significant unless otherwise specified. Statistical analyses were performed using SPSS 25 (IBMCorp) and R statistical software v4.0.2.

## Results

### Protective Effects of Metformin in an Acute Pneumonia/Sepsis Murine Model

**FIGURE 2:**
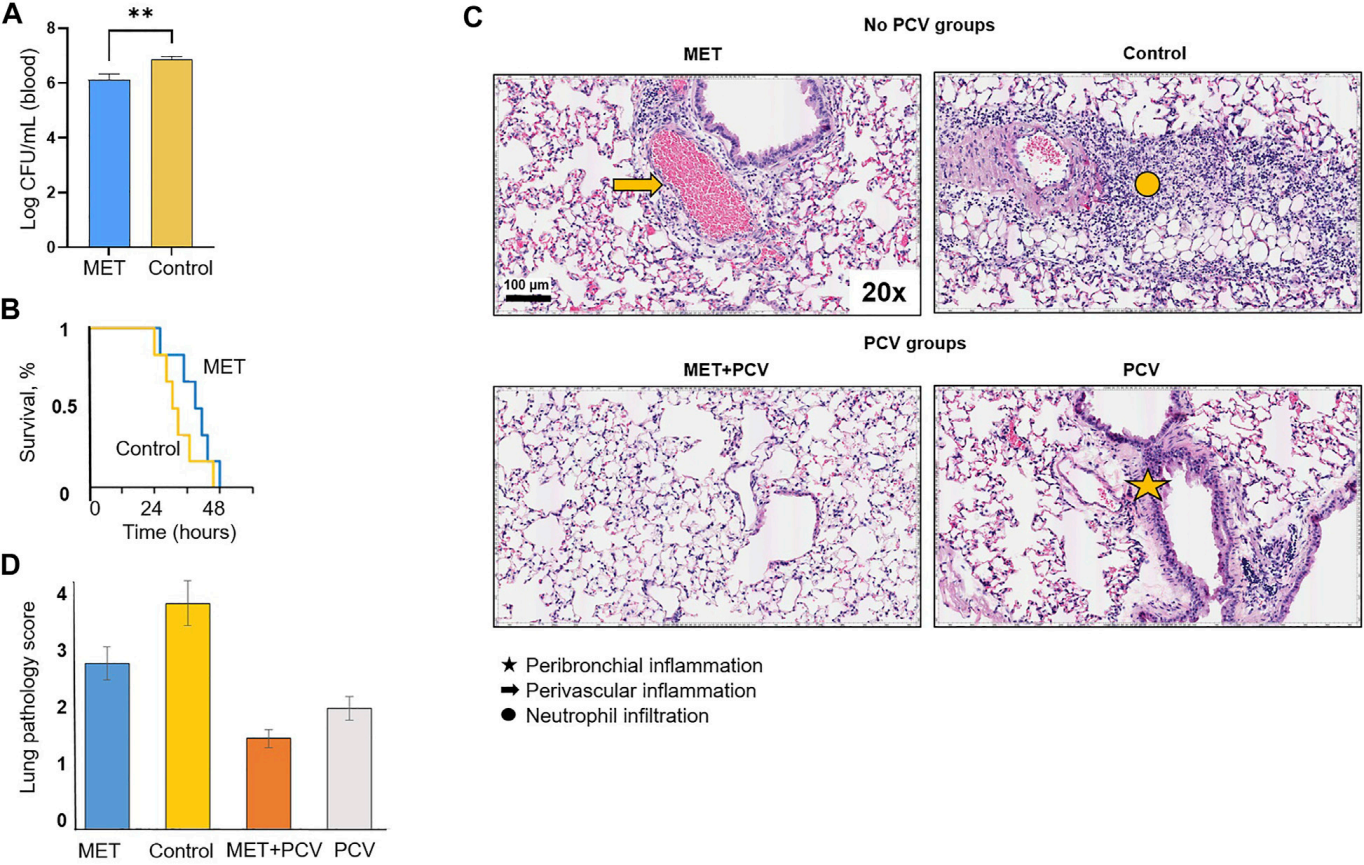
Protective effects of metformin in an acute pneumonia/sepsis murine model. **(A)** Bacterial burden of SPN in log colony forming units (CFUs) per mL volume of blood in MET only vs. control only groups at 24 h post SPN infection. *n* = 6 in each group. Depicted as mean ± SEM. **p < 0.01. **(B)** Survival curves for MET only vs. Control post intratracheal challenge of *Streptococcus pneumoniae* (SPN) serotype 4 strain TIGR4. Statistical analysis was performed using a Kaplan-Meier log rank test (*p =* 0.18). Mean time to death was compared using Students t-test. *n* = 6 in each group. **(C)** Hematoxylin and eosin stained lung sections after intratracheal inoculation of SPN (TIGR4 strain) from post-infection day 2 in no-PCV groups and post-infection day 7 in PCV groups. Comparisons of MET only (top left), control only (top right), MET + PCV (lower left), and control + PCV (lower right). Lung sections were examined for perivascular and peribronchial inflammation. *n* = 6 in each group. **(D)** Lung pathology score. Two lung sections were scored per mouse. Lung sections were scored blindly on a scale of 0–5 on the basis of peribronchial and perivascular inflammation, neutrophil infiltration, and alveolar thickening and consolidation. Measurements expressed as mean ± SEM. Statistical analysis was performed using Student’s t-test; * denotes *p* < 0.05 for MET only (blue) vs. Control only (yellow) groups; # denotes *p* < 0.05 for MET + PCV (orange) vs. PCV (gray) groups. Scale bar = 100 μm. PCV, pneumococcal conjugate vaccine; MET, metformin; SD, standard diet; SPN, *Streptococcus pneumoniae*; IT, intratracheal.

Four groups of SPN challenged mice were evaluated (Figure 1C). Animals in the MET-alone vs. control-alone group showed a 4-fold lower bacterial load in peripheral blood 24 hours-post SPN challenge (Figure 2A). This was accompanied by less infection-related morbidity (hypothermia, oxygen saturation, weight loss) in the MET group vs. controls (Supplementary Material). Mice in both the MET-alone and control-alone group died within 48 h. Survival analysis showed a trend for slower rates with MET-alone compared to control-alone mice (Figure 2B; *p* = 0.18). MET-alone mice had a longer mean time to death than the control-alone mice (41.3 vs. 33.3 h, respectively, *p* < 0.01). In contrast to the bacterial burden at 24 h post-infection, at 48 h post-infection there were no differences in the lung bacterial load between the MET-alone and control-alone groups (Supplementary Material). None of the mice receiving PCV had detectable levels (>10^2^ CFUs) of bacteria in blood at 24 h post infection and all survived to day 7; they were all sacrificed at this time point. Serum metformin levels were evaluated pre- and post-infection (at time of death). There were no significant differences between serum metformin levels of MET-alone vs. MET + PCV after 6 weeks on MET-diet (1,944 ng/ml vs. 1,396 ng/ml, *p* = 0.58); however, at time of death, the MET-alone group had undetectable serum levels (Supplementary Material).

Histopathological analyses of lung tissues showed extensive inflammatory cell infiltration in the pulmonary interstitial and alveolar spaces, with diffuse pulmonary hemorrhage, alveolar collapse, pulmonary interstitial edema, and extensive alveolar septal thickening in both MET-alone and control-alone groups (Figure 2C; top row). However, mice treated with metformin (MET-alone, MET + PCV) had less widespread inflammatory cell infiltration and alveolar collapse compared with respective control mice (Figures 2C,D).

**FIGURE 3:**
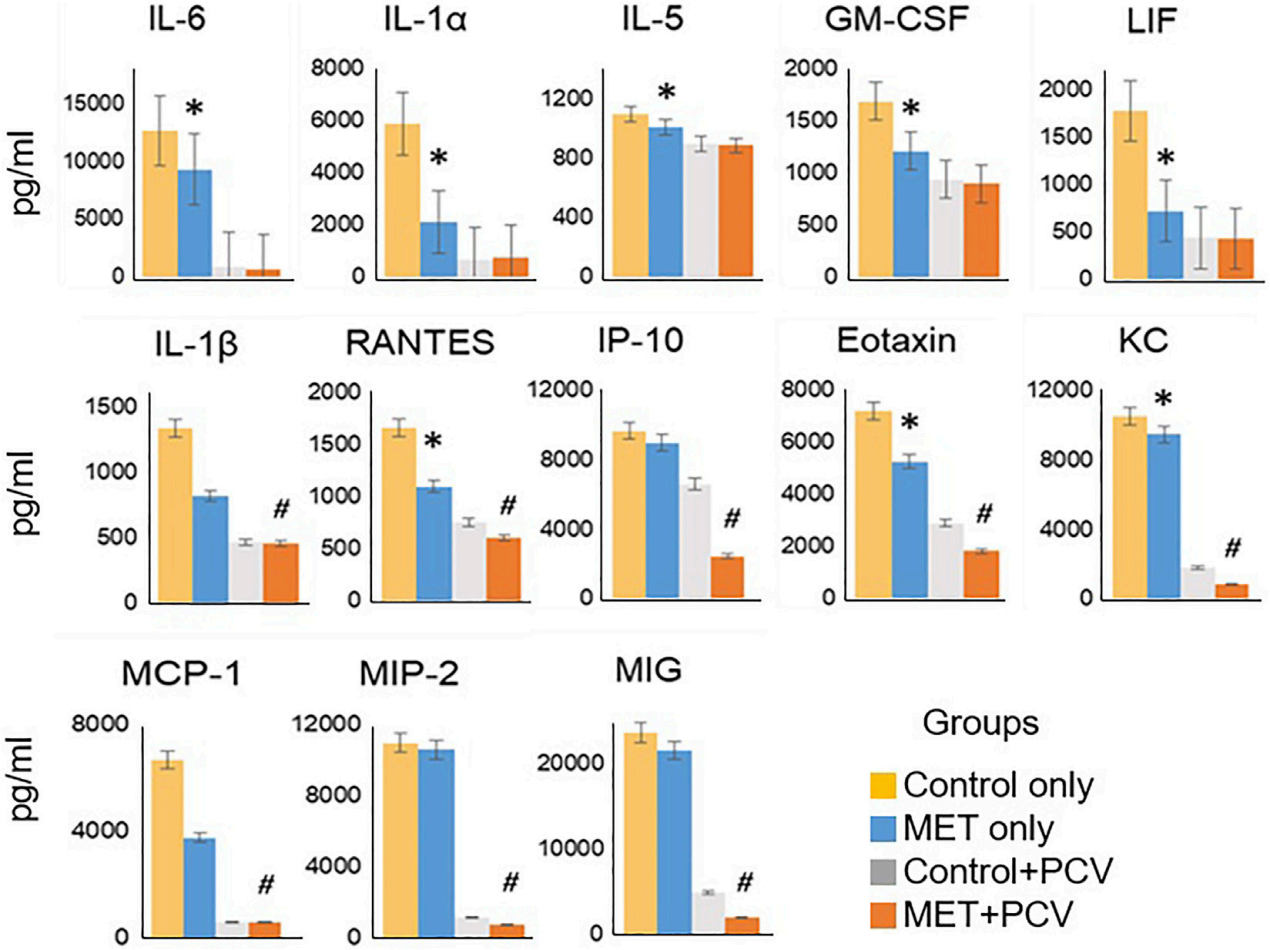
Metformin attenuates inflammatory levels in mouse lung tissue. Cytokines/chemokine analysis of lung homogenates of MET only vs. control only (no-PCV groups) at time of death post-SPN infection (all animals died within 48 h post-SPN infection); and MET + PCV vs. PCV only (PCV groups) at 7 days post-SPN infection. Data displayed for observed statistical differences between groups: * denotes *p* < 0.05 for MET only (blue) vs. Control only (yellow) groups; # denotes *p* < 0.05 for MET + PCV (orange) vs. PCV only (gray) groups. *n* = 3 in each group. Depicted as the mean ± SEM. PCV, pneumococcal conjugate vaccine; MET, metformin; Cntrls, controls; SPN, *Streptococcus pneumoniae*; IT, intratracheal.

Levels of IL-1α, IL-5, IL-6, IL-13, GM-CSF, LIF, and KC (CXCL1) were significantly lower in the lung homogenates of MET-alone vs. control-alone groups (all *p* < 0.05; Figure 3). Expression levels of IL-1β, RANTES (CCL5), IP-10, eotaxin (CCL11), KC, MCP-1, MIP-2, and MIG were significantly lower in MET + PCV vs. control + PCV group at 7 days post-infection (all *p* < 0.05; Figure 3).

Metformin levels in lung tissue were evaluated at time of death. In contrast to that of serum, detectable levels of metformin were observed in lung tissue of the MET-alone group, but were lower compared to that of the MET + PCV group (4.43 ng/ml per mg vs. 16.89 ng/ml per mg; *p* = 0.05; Supplementary Material). Consistent findings were observed comparing metformin levels in liver tissues of MET-alone vs. MET + PCV groups (Supplementary Material).

### Immunomodulatory Effects of Metformin During Acute SPN Challenge

**FIGURE 4:**
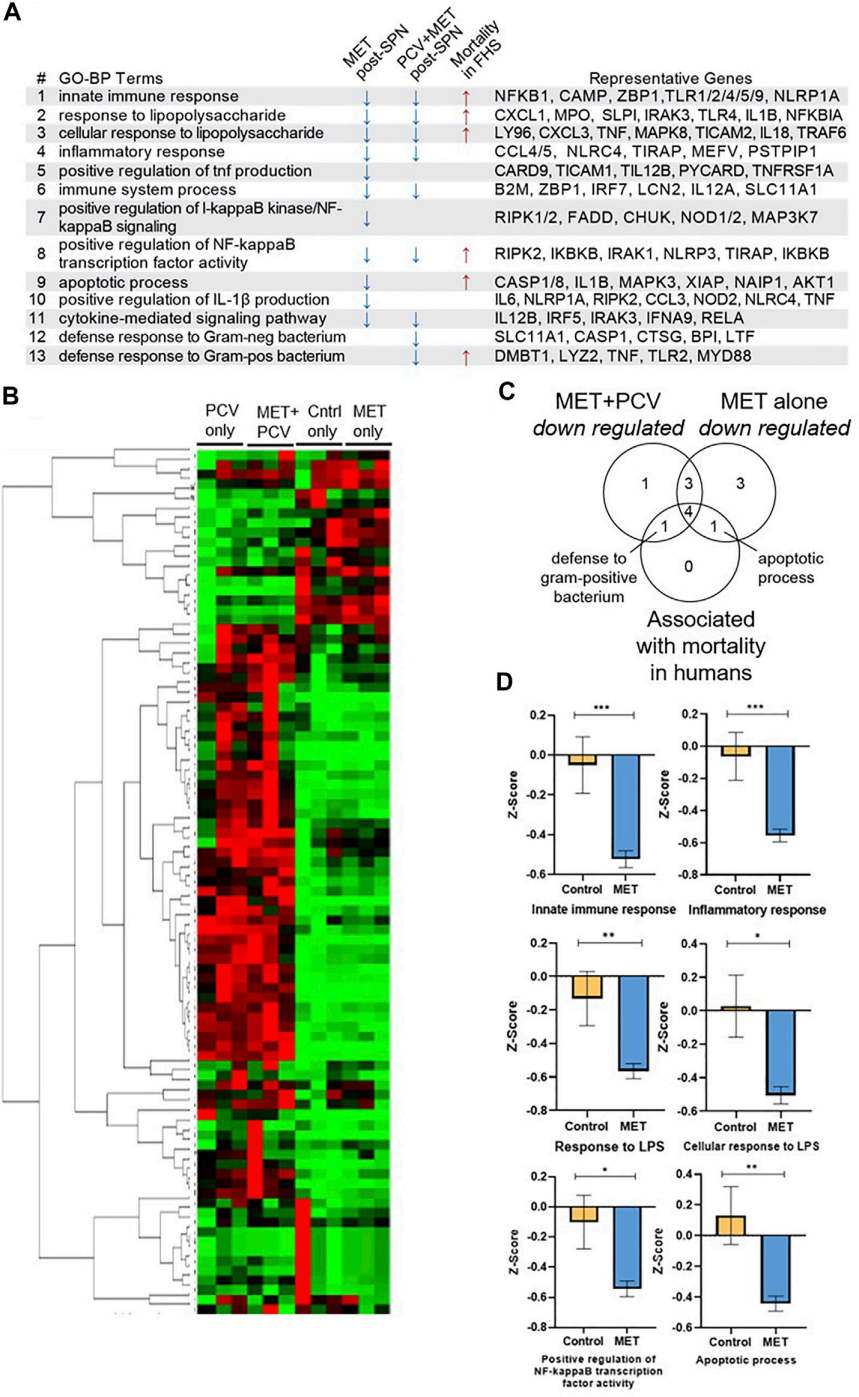
Immunomodulatory effects of metformin during acute SPN challenge. **(A)** Gene expression of lung tissue using RT2 Profiler PCR for Mouse Bacterial Response. GO-BP Terms associated with downregulated genes (>1.5 fold) in the MET-alone group vs. controls during acute SPN infection (day 2 post-SPN infection) and in the MET + PCV vs. control + PCV groups (day 7 post-SPN infection). GO-BP terms linked to six mortality-associated signatures associated with increased all-cause mortality hazards during COVID-19 and shorter lifespan in a 9-year follow up of the Framingham Heart Study Offspring Cohort (Lee et al., 2021). Right most column includes representative genes in each GO-BP Term. **(B)** Clustogram of gene expression of lung tissue between four groups performed on GeneGlobe (Qiagen) using double delta C_T_ method. **(C)** Venn diagram of shared and unique GO-BP terms that were either 1) downregulated by MET only vs. controls, 2) downregulated MET + PCV vs. PCV only groups, and/or 3) associated with mortality associated signatures found in COVID-19 and FHS human cohorts. **(D)** Comparison of gene-expression scores (z-scores) between MET only vs. Controls only for GO-BP terms representative of mortality associated signatures COVID-19 and FHS human cohorts. Measurements expressed as mean ± SEM. *n* = 3 in each group. ***p < 0.001; **p < 0.01; *p < 0.05. GO-BP term, gene ontologic biological processes; PCV, pneumococcal conjugate vaccine; MET, metformin; Cntrls, controls; SPN, *Streptococcus pneumoniae*; FHS, Framingham Heart Study Offspring Cohort; LPS, lipopolysaccharide.

The abovementioned findings suggested that treatment of mice with metformin before SPN infection may mitigate development of a severe inflammatory response during acute SPN infection. To identify potential protective mechanisms, we focused on GO terms linked to biological processes (GO-BP terms) that were differentially expressed in the metformin-fed mice. In the comparison of MET-alone vs. control-alone mice, down regulated genes (>1.5 fold) were linked to 11 GO-BP terms overrepresented in the MET-alone group (Figure 4A). These GO-BP terms related to inflammation including innate immune and inflammatory responses (Figure 4A; GO-BP terms #1, 4, 5, 6), lipopolysaccharide response (#2, #3), activating the NF-κB pathways (#7, #8), IL1β production (#10), and apoptosis (#9) (Figure 4A). Expression levels of the genes in the GO-BP terms were metricized (z-scores) and compared between animal groups. Z-scores were lower in MET-alone vs. control-alone groups. Notably, we did not identify pathways associated with genes whose expression levels were upregulated in the lung tissue from MET-alone vs. control-alone mice. Unsupervised hierarchical clustering of gene expression levels showed that samples from each group cluster together (Figure 4B).

Congruent results of lower inflammation were observed when comparing lung gene expression profiles of MET + PCV vs. control + PCV groups obtained 7 days post SPN challenge (Figures 1C,D). None of the mice had detectable levels of bacteria from lung homogenates, indicating that at day 7 they were in a post-acute bacteremic phase. Nine GO-BP terms were overrepresented in MET + PCV vs. control + PCV groups; seven of these are shared with the MET-alone observed during acute pneumonia, and two are uniquely associated with MET + PCV group (Figure 4C). Expression levels of genes in these GO terms were downregulated in the MET + PCV vs. control + PCV group.

### Metformin Attenuates Gene Expression Signatures Associated With Mortality

We recently developed gene expression (transcriptomic) signatures that tracked immunological resilience during COVID-19 and as well as in non-COVID-19 contexts (Lee et al., 2021). We identified two categories of transcriptomic signatures. The first category were survival-associated signatures comprising genes linked to immunocompetence; higher expression of these genes associated with increased survival in patients with COVID-19 and longevity in persons without COVID-19 (in the Framingham Heart Study). The second category were mortality-associated signatures comprising genes linked to inflammation; higher expression of these signatures associated with increased all-cause mortality hazards during COVID-19 and shorter lifespan (Lee et al., 2021). Notably, six of the mortality-associated signatures were also attenuated in the MET-alone vs. control-alone groups (Figures 4A,C; GO-BP terms #1, #2, #3, #8, #9, and #13). Expression levels of these six signatures were lower in the MET-alone vs. control-alone group (Figure 4D), suggesting that signatures that associate with shorter lifespans in humans are lower in metformin-fed mice in an acute infection model.

### Metformin Treatment Enhances SPN Antibody Responses

**FIGURE 5:**
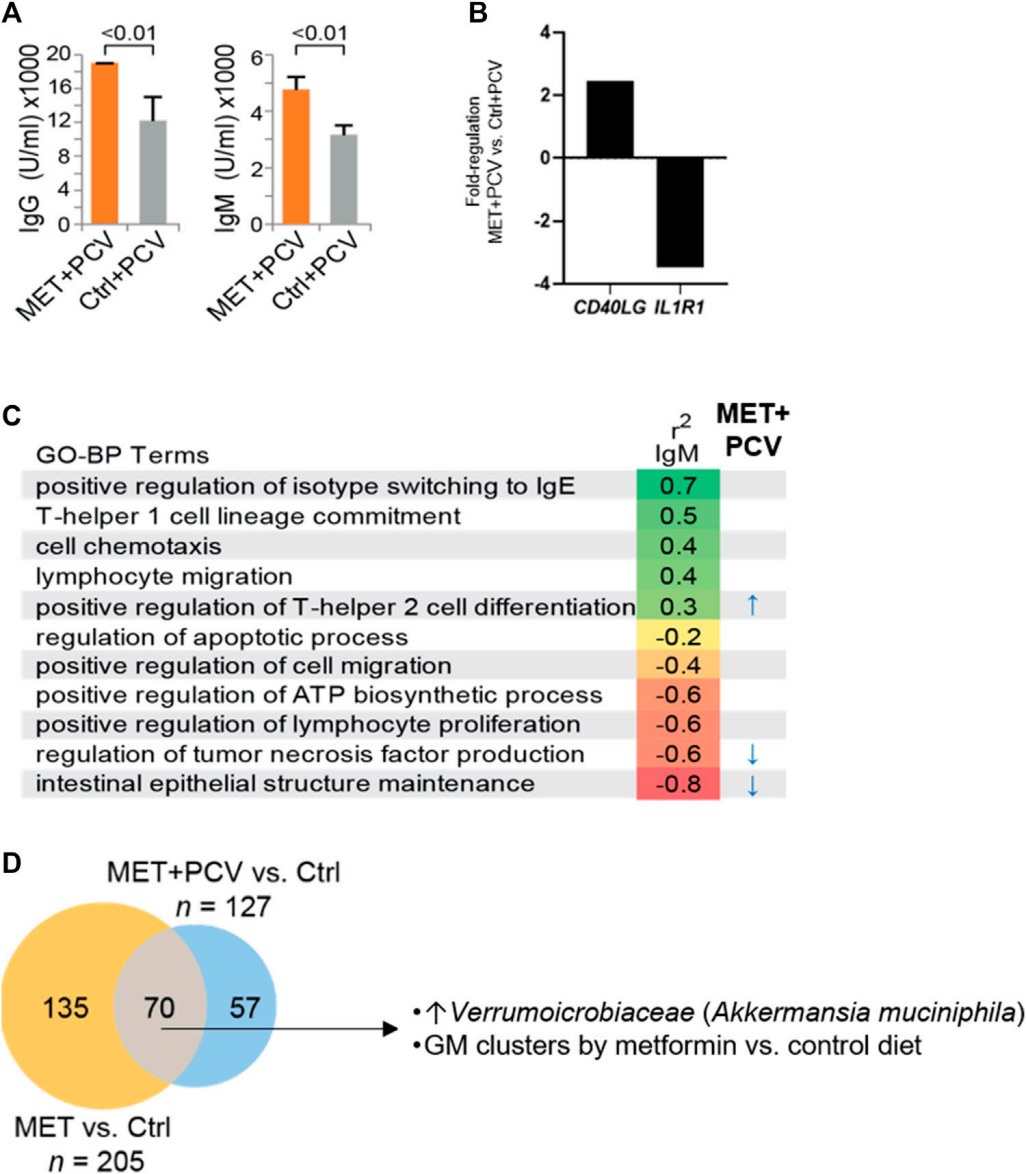
Metformin treatment enhances antibody responses and modulates gut microbiome. **(A)** SPN anti-capsular antibody levels of MET + PCV vs. Control + PCV at 2 weeks after PCV13. Left, IgG, Right IgM. *n* = 6 in each group. **(B)** Fold difference of a representative upregulated gene (*CD40LG*) and downregulated gene (*IL1R1*) from splenic cells of MET + PCV vs. Control + PCV groups. **(C)** SPN anti-capsular IgM levels and GO-BP terms (z-score) correlations performed using Spearman’s correlation coefficient (*p*) using an FDR of *p* < 0.05. GO-BP term scores significantly associated with MET + PCV vs. Controls + PCV indicated (right). *n* = 3 in each group. **(D)** Comparison of gut microbiomes composition (OTUs) between MET + PCV and MET only groups to the same control group identifies microbes consistently shifted by both metformin groups compared to controls (*n* = 6 in each group). Details of diversity and abundance are depicted in the supplement. GO-BP term, gene ontologic biological processes; PCV, pneumococcal conjugate vaccine; MET, metformin; Ctrls, controls; SPN, *Streptococcus pneumoniae*; FHS, Framingham Heart Study Offspring Cohort; OTU, operational taxanomic units; GM, gut microbiome.

Next, we examined whether metformin influenced humoral responses, i.e., antibody levels in mice receiving PCV. MET + PCV vs. control + PCV mice had significantly higher levels of SPN anti-capsular IgG (18,869 U/ml vs. 11,866 U/ml; *p* < 0.01) and IgM (4,760 U/ml vs. 3,160 U/ml; *p* < 0.01) (Figure 5A). Post SPN infection, MET + PCV vs. control + PCV mice sustained higher anti-capsular IgM levels (6,243 U/ml vs. 4,857 U/mL, *p* = 0.03). In splenic tissue, MET + PCV vs. control + PCV mice showed two significant gene expression differences: 1) higher levels of *CD40L*, a critical for T cell activation gene that plays a key role in generation of SPN anti-capsular IgG and IgM responses (Jeurissen et al., 2002), and 2) lower levels of *IL1R1*, the receptor for IL-1, a key inflammatory cytokine (Dinarello, 2011) (Figure 5B).

SPN anti-capsular IgM levels positively correlated with z-scores for the GO-BP terms of isotype switching to IgE, T-helper 1 cell lineage, cell chemotaxis, lymphocyte migration, and positive regulation of T-helper two cell differentiation (Figure 5C; r^2^ IgM). Whereas, SPN anti-capsular IgM levels negatively correlated with z-scores of GO-BP terms of inflammatory responses (intestinal epithelial structure maintenance, regulation of TNF), regulation of lymphocyte proliferation, and apoptosis.

Next, we examined whether the z-scores of the GO-BP terms that correlated with SPN IgM levels differed in MET + PCV vs. control + PCV groups. The GO-BP term related to T-helper two cell differentiation (representative genes: *IL18, NLRP3, IL4RA*) was positively correlated with higher SPN IgM levels, whereas the GO-BP term related to intestinal epithelial structure (*TLR4, IL17A*) and regulation of TNF production (*MYD88*) negatively correlated with SPN IgM levels (Figure 5C). Many of metformin’s effects are attributed to its effects on AMPK pathways (Musi et al., 2002; Kulkarni et al., 2020). However, we found no differences in levels of activated p-AMPK between MET + PCV vs. control + PCV groups (Supplementary Material).

To corroborate our gene expression findings, we evaluated publicly available gene expression databases. Obermoser et al. (2013) examined the correlation between the expression levels of gene modules with SPN antibody responses in middle-aged adults (GSE48762 in NCBI’s GEO). Strong negative correlations were observed on day 7 with expression of modules associated with inflammation (M3.2) and positive correlations were observed for expression of module M4.11 that associated with plasma cells (Supplementary Material). Another publicly available gene expression dataset (GSE57275) characterized the effects of metformin in a murine model of tuberculosis infection (Singhal et al., 2014). Our analysis showed that metformin treated mice associated with lower expression levels of the inflammatory module (M3.2) and associated with a trend for an increase in plasma cell (M4.11) module expression (Supplementary Material). Thus, our findings in these publicly available gene expression datasets support our findings that metformin modulates inflammation pathways.

### Metformin Modulates the Gut Microbiota

Gut microbiome dysregulation has been implicated in major conditions (e.g., obesity, T2DM, cancer) and shown to promote inflammation and gut permeability (Thevaranjan et al., 2018). Emerging studies have shown metformin’s immunometabolic role may relate to shifts in gut microbiota composition (Kulkarni et al., 2020; Forslund et al., 2015). We observed that metformin associated with lower expression of the GO-BP term related to intestinal epithelial structure maintenance that negatively correlated with SPN IgM production. We therefore hypothesized that metformin may attenuate inflammatory pathways by modulating the gut microbiome. We evaluated microbiome profiles of the four mice groups across five time-points (baseline, 6, 9, and 10 weeks, Figure 1C). There were no significant differences in bacterial diversity between time-points within and between groups using Shannon diversity metrics (Supplementary Material). We compared the aggregated differential abundance of microbes between metformin-treated groups vs. controls to identify 70 microbes that were consistently shifted by metformin (Figure 5D). At the order level, *Verrumoicrobiaceae*, *Lactobacillales, Erysipelotrichales,* and *Coriobacteriales* were significantly increased in mice gut microbiomes of metformin groups compared to controls (Figure 5D). This included a 5-fold higher abundance in the Verrucomicrobia, *Akkermansia muciniphila,* previously implicated with beneficial effects on intestinal integrity and longevity (Bárcena et al., 2019) (Figure 5D, Supplementary Material). Principal component analysis suggests that metformin treatment groups can be nearly all separated on PCo1 explaining 29% of the variability (Supplementary Material). Hierarchical clustering shows that baseline samples clustered together, however groups clustered together by diet group across all time points (Supplementary Material).

## Discussion

While chronologic age is associated with a deterioration in immunological resilience linked with the passage of time, this deterioration is also observed when experiencing environmental (e.g., infectious) triggers/challenges that can occur at any age (Lee et al., 2021; Marconi et al., 2022). Thus, both sources contribute to the wide heterogeneity in the capacity of humans to preserve immunological resilience throughout life. Conceivably, some younger persons have an increased susceptibility to erode immunological resilience prior to and/or in response to antigenic stimulation that may predispose to accelerated onset of erosion of immunologic resilience during adulthood. Herein, we assessed metformin as a possible *immunoprotectant* for attenuation of the decline in immunological resilience that may occur during an acute infectious challenge. As a representative challenge, we focused on SPN infection, as it is associated with considerable morbidity and mortality (Feikin et al., 2000).

During acute SPN sepsis, metformin for 10 weeks pre-challenge associated with lower infection related morbidity including bacterial burden and lung pathology. Correspondingly, this was associated with an attenuated inflammatory response, indicated by lower levels of pro-inflammatory markers and expression of genes related to the LPS response, NF-κB pathway, IL1β production, and apoptosis, all established innate/inflammatory signaling pathways. These findings are consistent with prior studies describing metformin’s anti-inflammatory effects (Zhou et al., 2001; Berstein, 2012; Martin-Montalvo et al., 2013; Ursini et al., 2018; Titov et al., 2019; Kulkarni et al., 2020; Xiao et al., 2020). Notably, metformin associated with lower levels of gene expression signatures previously shown to associate with mortality in COVID-19 and shorter time to death in the Framingham Heart Study Offspring cohort (Lee et al., 2021). Thus, these findings shed insight into mechanisms underlying metformin’s potential life-extending effects (Martin-Montalvo et al., 2013; Kulkarni et al., 2020). Moreover, metformin intake mimicked chronic use in humans (vs. adjunct during an acute infection) suggesting that its beneficial effects on immunological resilience might have been imparted prior to the onset of an acute infection challenge. Consistent with these inferences, metformin use prior to admission among persons with type 2 diabetes has been associated with a nearly 40% lower mortality rate (Liang et al., 2019). These observations may be attributable to metformin mitigating age-associated chronic inflammation or response to acute inflammatory stimuli.

We identified potential mechanisms by which metformin associated with enhanced SPN antibody levels after PCV administration. First, metformin associated with higher gene expression levels of *CD40LG,* critical in T and B cell signaling for germinal center formation, isotype class switching, and for the production of SPN anti-capsular IgG and IgM responses (Jeurissen et al., 2002). Second, metformin attenuated gene expression levels of two gene pathways that negatively correlated with SPN IgM levels. This included processes linked to TNF production, a finding that is consistent with prior studies which found that elevated TNF levels impaired anti-pneumococcal immunity when old mice were challenged with SPN (Puchta et al., 2016). The other process was linked to intestinal epithelial regulation suggesting the role of gut microbiome on systemic inflammation (Thevaranjan et al., 2017; Seidel and Valenzano, 2018; Bosco and Noti, 2021). To expound on this observation, we compared the gut microbiomes in metformin vs. control mice. Metformin significantly increased the abundance of *Akkermansia muciniphila*. This finding is notable, as loss of this species has been associated with age and is linked with impaired intestinal barrier function, obesity, and insulin resistance (de la Cuesta-Zuluaga et al., 2017; Bárcena et al., 2019). In addition, *A. muciniphila* has been previously implicated in playing a role in regulating T-cell responses for antibody production (Ansaldo et al., 2019). Thus, the muted inflammation observed with metformin may be attributable to several concurrent mechanisms mediated directly and indirectly by its effects on the gut microbiome. Together, these findings suggest that metformin may enhance many aspects of the immune system to mitigate erosion of immunologic resilience.

This study has limitations. This study focuses on evaluation of a strategy to mitigate the loss of immunological resilience, with a focus on primary prevention using metformin (Figure 1A). For this reason, young adult mice were studied in a high-grade infection challenge model. However, we did not directly evaluate metformin for secondary prevention (i.e., later life), as the accretive effects of antigenic exposures experienced across the lifespan may also erode immunological resilience over time (Figure 1A). Subsequent studies are needed to explore the optimal time (age) to target for primary and secondary prevention as well as dosing in varied challenge models in different species. Herein, metformin administration of 6 weeks prior to vaccination was used on the basis of several prior studies including a murine study that demonstrated a 6-week course of an mTOR inhibitor that increased naïve lymphocytes, influenza vaccination responses, and extended life span (Chen et al., 2009). Moreover, in a murine model of *Legionella pneumophila*, a survival benefit was not observed when metformin was initiated at time of infection but only when metformin was started at least a week before and continued for more than 2 weeks after infection (Kajiwara et al., 2018). Consistent with these studies, our findings indicate that the disease mitigation effects of metformin in a SPN model may require several weeks of therapy prior to the challenge. Additionally, we had aimed to treat the animals for a duration that may align in humans with the duration required to observe metformin’s effects for chronic conditions (e.g., diabetes). Furthermore, the dose of the metformin-diet regimen we used were based on the studies of Martin-Montalvo et al. (2013) where the lower dose (used herein) was associated with 5.83% extension of mean lifespan in middle aged adult mice, whereas the higher dose associated significantly with shortened mean lifespan by 14.4% and renal failure. This dosing yielded concentrations of metformin lower than other studies targeting AMPK activation. Other studies have suggested metformin can exert immunomodulatory effects (i.e., NF-κB signaling pathway) using lower doses and independent of AMPK activation. For example, Cameron et al. found metformin’s suppression of NF-κB signaling activation at low chronic doses (0.05–0.25 mM) (Cameron et al., 2016). The effects of metformin at varying doses should be further characterized in specific tissues and cell populations to examine the long-term effects of metformin and role of AMPK, ideally in humans.

The preservation of immunological resilience is associated with survival, longevity, and resistance of infections (Lee et al., 2021). Thus, strategies to promote this preservation may mitigate the impact of its erosion, at any age, potentially augmenting health- and lifespan. Herein, adult mice that received metformin as primary prevention associated with features of immunologic resilience including attenuated SPN disease severity, lower inflammatory levels, and enhanced PCV vaccine responses. This may have implications for preventive strategies for prevention of an accelerated loss of immunological resilience.

## Data Availability

Data files supporting the results of this article will be made available by the authors without undue reservation.
