# Supplementary material for: Metformin Attenuates Inflammatory Responses and Enhances Antibody Production in an Acute Pneumonia Model of Streptococcus pneumoniae
Source: Front Aging. 2022 Apr 29;3:736835. doi: 10.3389/fragi.2022.736835 (PMC9261336; doi:10.3389/fragi.2022.736835)
Supplement: Supplementary file 1 [file DataSheet1.pdf]

Supplement

Metformin attenuates inflammatory responses and enhances antibody production in an acute pneumonia model of *Streptococcus pneumoniae*

Abbreviations: PCV, pneumococcal conjugate vaccine; MET, metformin; Ctrls, controls; SPN, *Streptococcus pneumoniae*; IT, intratracheal; GO-BP term, gene-ontologic biological processes; OTU, operational taxanomic units; GM, gut microbiome.

1. Safety and physiologic assessments 2
1.1. Metformin diet 2
1.2. Physiological responses in sepsis 2

2. Inflammation 3
2.1. Systemic inflammation. 3
2.2. Matrix of inflammation in lungs and liver. 4

3. Metformin concentration analysis using LC-MSMS 5

4. p-AMPK levels 6

5. Genomic correlates of SPN antibody responsiveness 7

6. Effects of metformin on mice gut microbiota 8
6.1 Diversity and statistical analyses 8
6.2 Differential abundance between metformin vs. no metformin groups. 9
6.3. Hierarchical clustering of metformin groups and timepoints. 10

7. Supplemental Figures 2
Figure S1 2
Figure S2 2
Figure S3 3
Figure S4 6
Figure S5 7
Figure S6 8-10

8. Supplementary Tables. 4
Table S1. Differential levels of inflammation in MET and PCV groups by lungs vs. liver 4
Table S2. Metformin concentration analysis using LC-MSMS by tissue 5

1. Safety and physiologic assessments

1.1 Metformin diet

Metformin was well tolerated and no deaths occurred prior to infection. MET fed mice did not lose weight and had comparable weight profiles to controls at time of vaccination (**Figure S1**). All weighed 19-26g at the beginning of the study and consumed average of 1.5g of diet daily up until infection.

Fig. S1

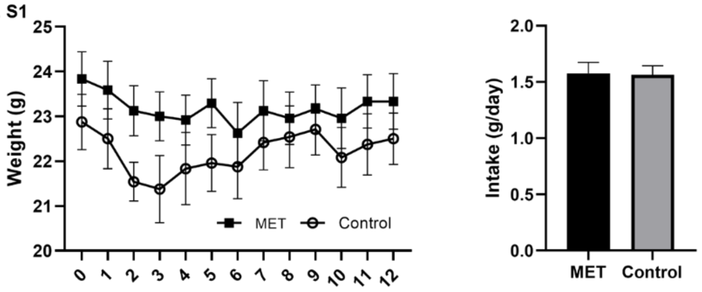

1.2. Physiological responses in sepsis

Unvaccinated mice became severely hypothermic with temperatures below 33°C compared with vaccinated mice (**Fig. S2**), higher proportion of the control group mice developed severe hypothermia vs. MET group. Low peripheral arterial oxygen saturation was noted within 12 hours among nonvaccinated mice (-7.2% MET only vs. -10.9% Control; p <0.01), whereas none of the vaccinated mice developed lower oxygen saturations. Significant weight loss was observed among unvaccinated groups 24-48 hours after SPN infection, while vaccinated mice increased body weight over the 7 days (**Fig. S2**).

Fig. S2

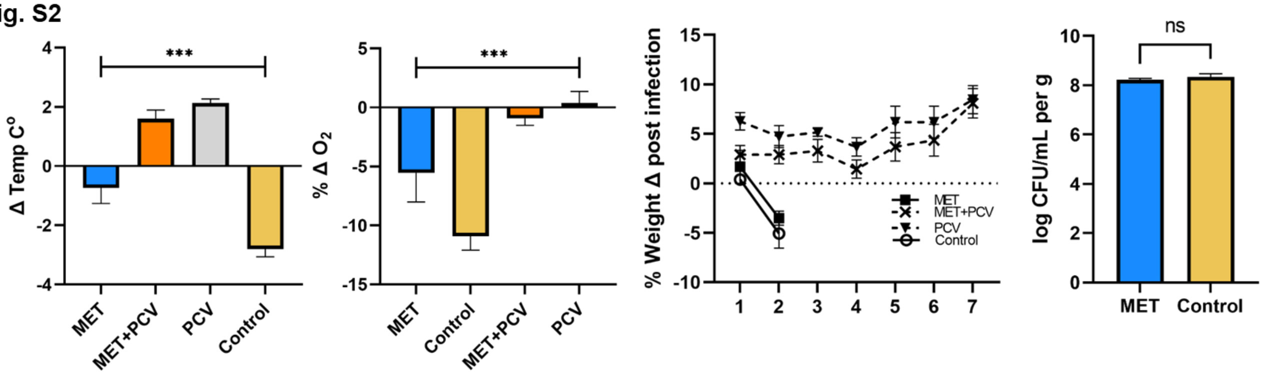

2.1 *Systemic Inflammation*

Inflammatory levels in the plasma were not significantly different between MET-fed mice compared to the controls at end of experiment (**Fig. S3**).

Fig. S3

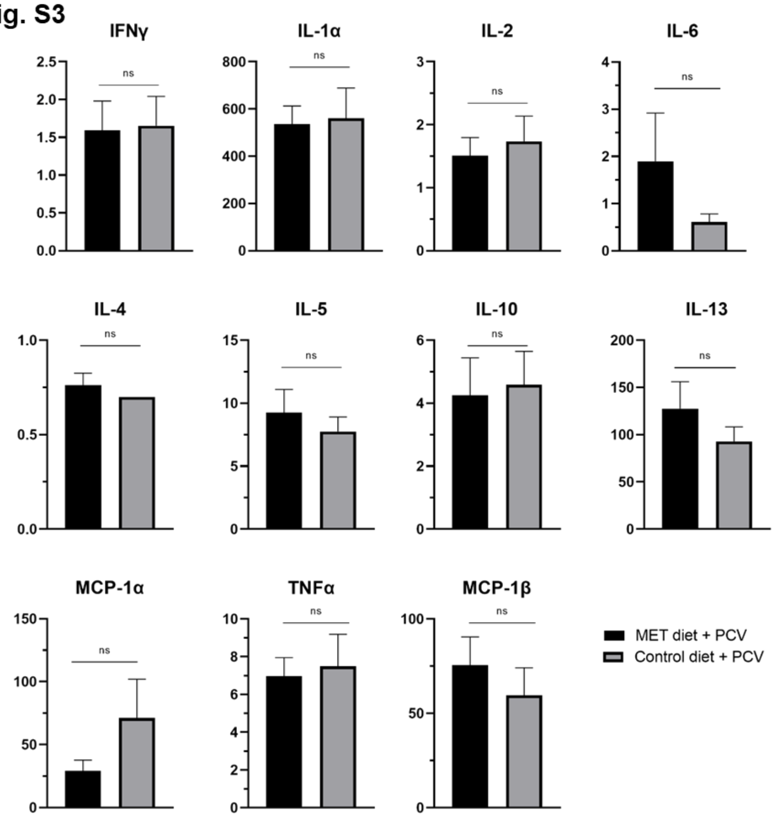

2.2 Matrix of inflammation in lungs and liver

Table S1. Differential levels of inflammation in metformin and PCV groups by inflammation in lungs vs. liver

Groups	Lower LUNG cytokines	Lower LIVER cytokines	Higher LIVER cytokines
**MET** vs. Control	IL-1 alpha, IL-5, IL-6, IL-13, GM-CSF, LIF, KC	MIP-2, RANTES	
**PCV group** vs. No PCV group	IL-1 beta, IP-10, eotaxin, KC, RANTES	IL-6, IL-9, IP-10, G-CSF, eotaxin, GM-CSF, LIF, KC, MIP-1 alpha, MIP-1 beta, M-CSF, MIP-2, MIG, RANTES	IL-1 alpha, IL-1 beta, IL-2, IL-4, IL-10, VEG-F
**MET+PCV** vs. Control+PCV	IL-1 beta, IP-10, eotaxin, KC, RANTES	IL-1 alpha, IL-2, IL-4, IL-6, IL-9, IL-10, G-CSF, GM-CSF, LIF, MIP-1 beta, MIP-2, RANTES	IL-1 beta, IP-10, eotaxin, KC, MIP-1 alpha, M-CSF, MIG, VEG-F

Reference group is bolded.

3. Metformin concentration analysis using LC-MSMS

Metformin levels was evaluated in serum were evaluated after 6 weeks of metformin and after SPN infection (at time of death in unvaccinated mice or 7 days after infection in vaccinated mice). A developed surrogate serum standard curve (5-3,000 ng/mL MET) that included a select internal standard (Propranolol, Sigma Aldrich) with the standard compound, metformin (Sigma Aldrich) with liquid solvent precipitation extraction system was used to remove the target compounds from serum matrix. The subsequent separation by ultra high pressure liquid chromotagraphy (Waters, UPLC, H-class system; analytical column C18 2.1x50mm column) of the target compounds presented ions that can be ionized by atmospheric pressure electrospray ionization for detection and measurement by LC-MSMS (Waters, Zevo TQD). After MS optimization for each compound was determined, the LC-MSMS parameter mode of (ESI+, MRM) was selected for mass-to-charge ratio (m/z) for the following parent to daughter transition ions. Ionized compounds (metformin, ESI+, MRM of parent to daughter, 130.00-59.980 m/z) and (I.S. Propranol, 260.18-116.09 m/z). Data were analyzed using the Waters Target Lynx program.

Freshly harvested mouse whole lung and liver tissue were flash frozen with liquid nitrogen. A target wet weight of 50 mg was used. Samples were transferred to a Fast-Prep bead homogenization tube and 1 mL of ethanol organic extraction solvent with the IS. The tubes were centrifuge for 10 minutes at 10,000 rpm at 40C and dried using a speed-vac system. Each sample was reconstituted with 50 uL of 100% acetonitrile.

Table S2. *Metformin concentration analysis using LC-MSMS by tissue*

	**MET-alone**	**MET+PCV**	***P***
**Serum** (ng/mL) post 6 weeks	1944	1396	0.58
**Serum** (ng/mL) post SPN infection	ND	1697	~
**Lung** (ng/mL per mg)	4.43	16.89	<0.05
**Liver** (ng/mL per mg)	2.28	28.18	<0.01

4. p-AMPK levels

Mouse AMPK (Phosphorylated Adenosine Monophosphate Activated Protein Kinase) ELISA Kit was used according to manufacturer's instructions on splenic cells. A frozen cell suspension containing ~1x10^6 cells were thawed on ice. The sample volumes were centrifuge to pellet the cells and the supernatant removed. A wash volume of 250uL of per-cooled PBS was used to resuspend the cell gently and again centrifuge to pellet the cells. All cell samples were suspended in 250uL of PBS and allowed to complete a multiple pellet freeze thaw process until all cells are fully lysed. The sample tubes centrifuge for 10 minutes at 1500xg at 4C. The ELISA Kit assay test procedure is based on the Sandwich-ELISA principle. The micro plate has been pre-coated with an antibody specific to Mouse AMPK. The standards and samples added to the plate wells and combined with the specific antibodies. Then a biotinylated detection antibody specific for mouse AMPK and Avidin-Horseradish Peroxidase conjugate are added to each well and incubated. Free components are washed and a substrate solution is added. Wells that contain mouse AMPK, biotinylated detected antibody and Avidin-HRP conjugated appear blue. The optical density (OD) is measured spectrophotometrically at a wavelength of 450nm. The concentration of the mouse p-AMPK in the study samples were calculated by comparing the OD of the samples to the standard curve.

Fig. S4 p-AMPK on splenocytes

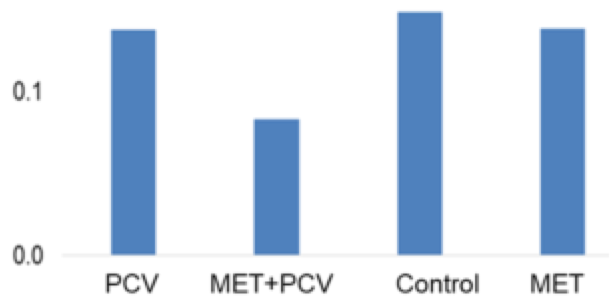

5. Genomic correlates of SPN antibody responsiveness

Obermoser et al.[1] evaluated the correlation between the expressions of well-characterized gene modules with SPN antibody responses post-vaccine in healthy controls. Strong negative correlations were observed on day 7 with expression of modules associated with inflammation (M3.2, M4.2, and M4.13) and positive correlations were observed for expression of module M4.11 that correlated with plasma cells. Fig. S5A (adapted from Obermoser et al.) depicts the correlations of M3.2 and M4.11 module expression with by SPN serotype on day 7 (blue and red: negative and positive significant correlations). M3.2 module is enriched for TLR genes. We evaluated this publicly available gene expression dataset (GSE48762 in NCBI's GEO)[2], focusing on the kinetic profile of the inflammation (M3.2) and plasma cell-associated (M4.11) modules that correlated with SPN antibody responsiveness. The kinetics of the expression of the inflammatory modules showed a consistent pattern: levels increased on day 1 and reached baseline levels on day 7 (Fig. S5B). In contrast, the plasma cell module expression increased on day 7 and reached baseline levels by day 21.

Fig. S5

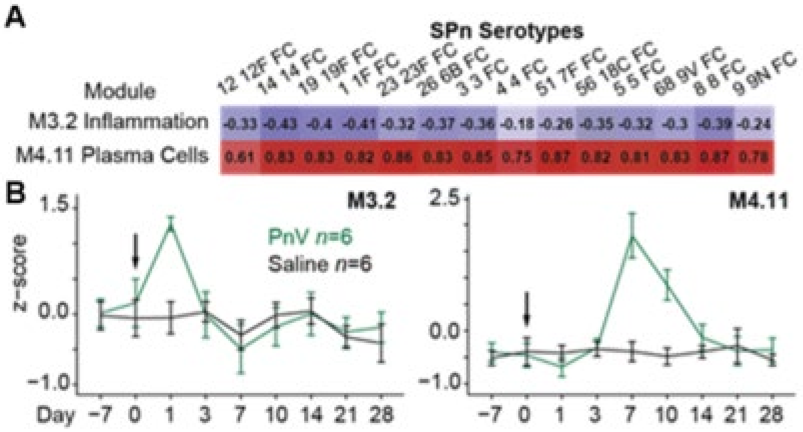

6. Effects of metformin on mice gut microbiota

6.1 Diversity and statistical analyses

Diversity and statistical analyses. The alpha diversity (within-population diversity) of samples will be measured using counts of observed species, the chao1 estimator for species richness, and the Shannon diversity index (**Fig. S6A**, left), which estimates total diversity taking into account both species richness and evenness. Beta diversity (diversity between populations) will be calculated using UniFrac distances between samples (based on the relative abundance of species), and visualized using principal coordinates analysis. Principal component analysis suggests that metformin treatment groups can be nearly all separated on PCo1 explaining 29% of the variability (**Fig. S6A**, right).

Fig. S6

A

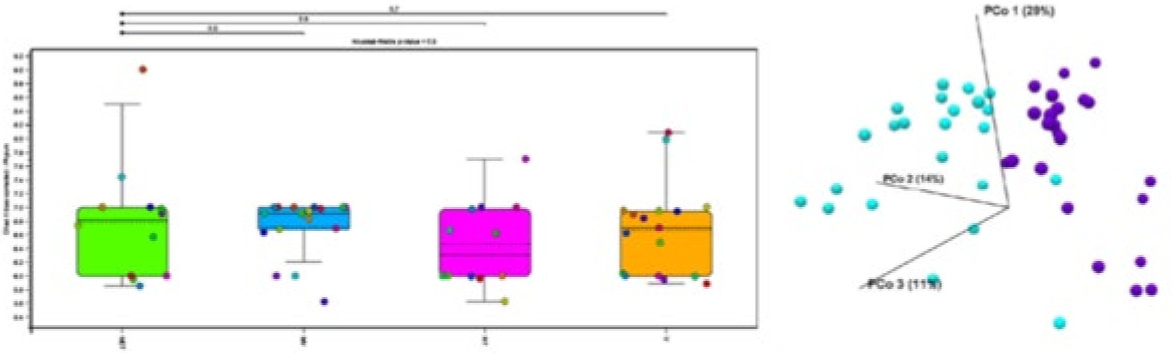

Fig. S6

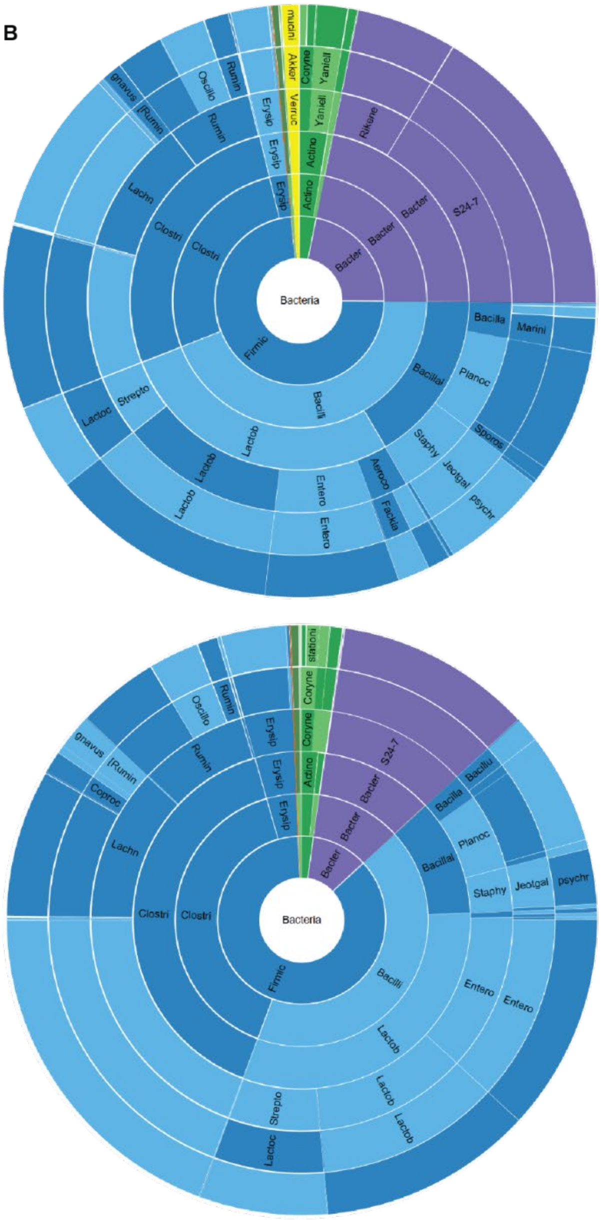

6.2 Differential abundance between metformin vs. no metformin groups. Sunburst plots of metformin group (*top*) vs. no metformin group (*bottom*) colored by order. At the order level, there was higher abundance of Verrumoicrobiaceae (yellow) with metformin.

Fig. S6

C

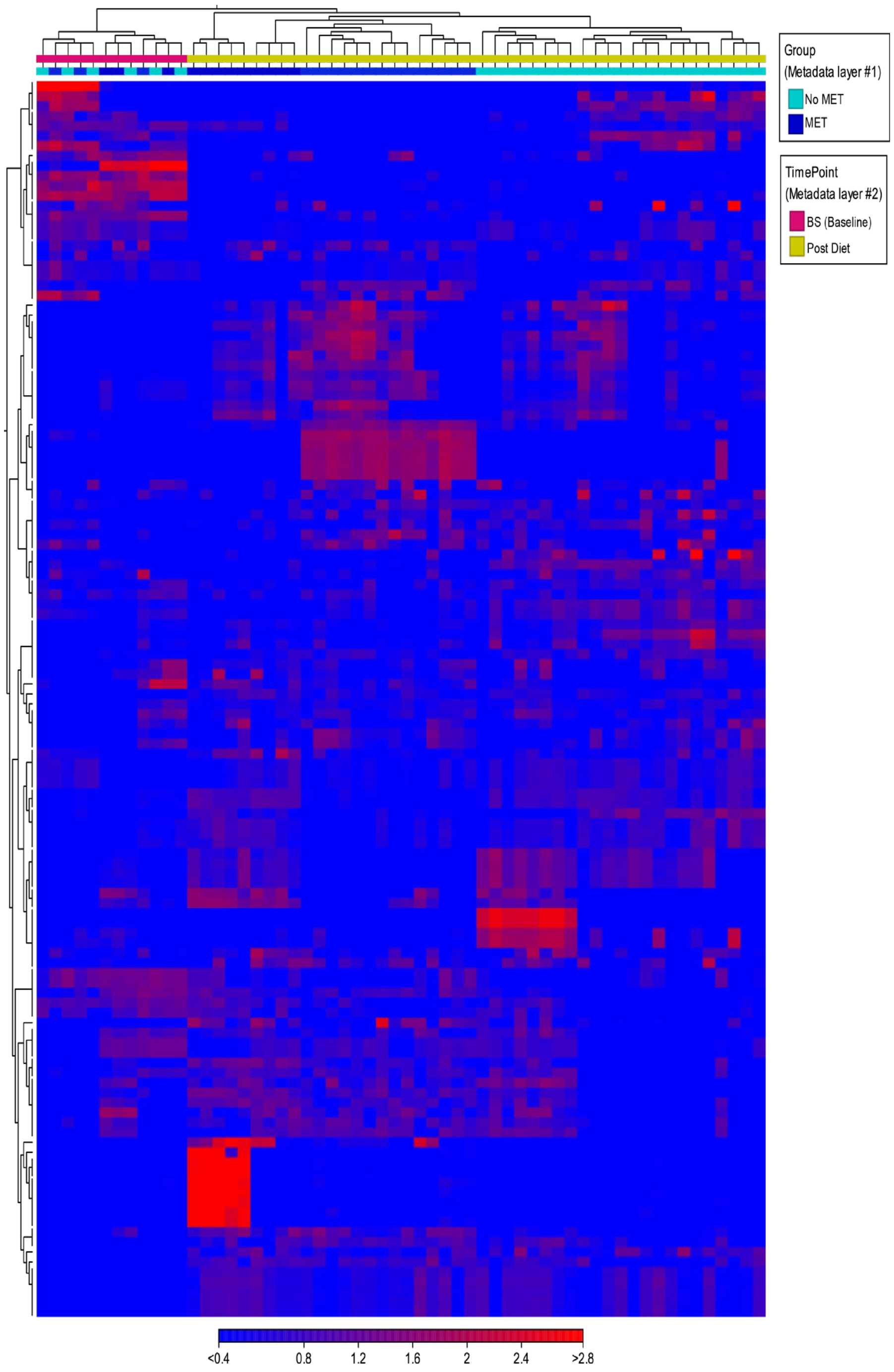

6.3. Hierarchical clustering of metformin groups and timepoints. Clustering of OTUs by metformin (light blue) vs. controls (dark blue) groups (layer 1) and by sample time point of baseline (before metformin diet allocation, red) vs. timepoints after diet allocation (post diet, yellow).

References:

1. Obermoser G, Presnell S, Domico K, et al. Systems scale interactive exploration reveals quantitative and qualitative differences in response to influenza and pneumococcal vaccines. *Immunity.* 2013;38(4):831-844.
2. Singhal A, Jie L, Kumar P, et al. Metformin as adjunct antituberculosis therapy. *Sci Transl Med.* 2014;6(263):263ra159.
